# Der „Surgical Track“ – innovative Ansätze gegen den Nachwuchsmangel in der Chirurgie

**DOI:** 10.1007/s00104-023-02029-y

**Published:** 2024-01-25

**Authors:** Rabi R. Datta, Joana Bohle, Thomas Schmidt, Hans Fuchs, Christiane J. Bruns

**Affiliations:** Klinik für Allgemein‑, Viszeral-, Tumor- und Transplantationschirurgie, Universitätsklinik Köln, Kerpener Str. 62, 50937 Köln, Deutschland

**Keywords:** Frauen in der Chirurgie, Junge Chirurgie, Virtual Reality, Robotische Chirurgie, Chirurgische Lehre, Female surgeons, Young surgery, Virtual Reality, Robotic Surgery, Surgical education

## Abstract

Die Chirurgie steht vor bedeutenden Herausforderungen, die sowohl aus Veränderungen in der medizinischen Ausbildung als auch aus der sinkenden Attraktivität des chirurgischen Berufswegs für angehende Ärzt:innen in der westlichen Welt resultieren. So haben sich die Erwartungen der Studierenden an ihren zukünftigen Arbeitsplatz geändert, wobei Themen wie Unsicherheiten in der beruflichen Planung, eine unausgewogene Work-Life-Balance sowie eine fehlende Vereinbarkeit von Familie und Beruf zunehmend relevant sind. Auch der Eintritt der Generation Z in das Berufsleben wird Auswirkungen auf die Chirurgie haben. Obwohl Frauen den größten Anteil der Absolvent:innen ausmachen, entscheiden sich nur wenige von ihnen für eine chirurgische Laufbahn. Der daraus resultierende Nachwuchsmangel wird die medizinische Versorgung in deutschen chirurgischen Kliniken negativ beeinflussen. Ein intensiver Wettbewerb um Talente zeichnet sich bereits in allen medizinischen Fachgebieten ab. So ergreifen Kliniken verschiedene Maßnahmen gegen den bevorstehenden Personalmangel, wie z. B. „Summer Schools“ oder Stipendien mit Arbeitsverpflichtungen. Darüber hinaus werden regionale Fördergesetze etabliert. Da insbesondere ein abnehmendes Interesse an chirurgischer Weiterbildung im Studienverlauf zu verzeichnen ist, ist zudem eine frühe Integration chirurgischer Fähigkeiten ins Medizinstudium entscheidend, um diesem Trend entgegenzuwirken. Aus diesem Grund haben wir den „Surgical Track“ entwickelt, der gezielt innovative Lehrkonzepte anbieten soll, um Studierende frühzeitig für das Fach Chirurgie zu begeistern. Dieser basiert auf den Säulen der Virtual Reality (VR) und der Robotik. Studierende können durch VR-Simulationen Operationen und Notfallszenarien trainieren sowie praktische Übungen mit Robotersystemen absolvieren. Qualitativ hochwertige Ausbildungskonzepte wie der „Surgical Track“ können dazu beitragen, Begeisterung für die Chirurgie zu fördern und gleichzeitig Wissen zu vermitteln, auch wenn ihr langfristiger Nutzen noch evaluiert werden muss. Durch virtuelle Simulationen, robotische Chirurgie und innovative Lehre erhalten Studierende Einblicke in die Viszeralchirurgie, die theoretisches Verständnis und praktische Erfahrung miteinander vereinen.

## Abstract (en)

Surgery faces significant challenges resulting from changes in medical education and the declining attractiveness of the surgical career path for aspiring doctors in the western world. For example, students’ expectations of their future workplace have changed, with issues such as career planning uncertainties, an unbalanced work-life balance, and a lack of compatibility of family and occupation becoming increasingly more relevant. The entry of Generation Z into the workforce will also impact surgery. Although women comprise the largest proportion of graduates only a few opt for a career in surgery. The resulting shortage of young surgeons will negatively impact medical care in German surgical units. Intense competition for talents is already emerging in all medical specialties. Thus, hospitals and academic centers are taking various measures to counteract the impending staff shortage, such as summer schools or scholarships with work commitments. Furthermore, regional funding laws are being established. In addition, as there is a declining interest in surgical training, particularly during the course of medical studies, early integration of surgical skills is crucial to counteract this trend. For this reason, we have developed the “surgical track”, designed to offer targeted innovative teaching concepts to get students attracted to surgery at an early stage. The “surgical track” is based on virtual reality (VR) and robotics. Students can practice operations and emergency scenarios through VR simulations and complete practical exercises with robotic systems. High-quality training concepts such as the “surgical track” can help to promote enthusiasm for surgery and impart knowledge at the same time, even if the long-term benefits still need to be evaluated. Through virtual simulations, robotic surgery and innovative teaching, students gain insights into visceral surgery that combine theoretical understanding and practical experience.

## Hintergrund

Die chirurgischen Disziplinen stehen derzeit vor bedeutenden Herausforderungen. So wurde im Juni 2023 der neueste Referentenentwurf der neuen Approbationsordnung veröffentlicht, der voraussichtlich ab dem 01.10.2027 in Kraft treten soll. Dieser Entwurf basiert auf dem „Masterplan 2020“, der wiederum auf dem Nationalen Kompetenzbasierten Lernzielkatalog Medizin (NKLM) aufbaut [1]. Das Hauptaugenmerk liegt hierbei auf der Verknüpfung theoretischer Grundlagen mit klinischen Inhalten und zwar von Beginn des Studiums an (vertikale und longitudinale Integration der Fächer). Die Aufteilung zwischen vorklinischem und klinischem Abschnitt wird hierdurch aufgehoben. Zudem soll eine interprofessionelle Ausbildung mit den Gesundheitsfachberufen gefördert werden. Viele Fachorganisationen, wie z. B. die von der DGCH (Deutsche Gesellschaft für Chirurgie) gegründeten Chirurgischen Arbeitsgemeinschaft Lehre (CAL), sehen dies als positive Entwicklung, da Studierende so besser auf die komplexe ärztliche Rolle und die spätere Berufsausübung vorbereitet werden können [2]. Allerdings wird in der zukünftigen medizinischen Ausbildung dem Fach Allgemeinmedizin und der ambulanten Patientenversorgung ein höherer Stellenwert eingeräumt. Dies wird sich durch eine erhöhte Präsenz in den Lehrinhalten während des Studiums und des Praktischen Jahres (PJ) zeigen. So ist geplant, das PJ in vier Abschnitte aufzuteilen, in denen neben den Fachgebieten der Inneren Medizin, Chirurgie und dem Wahlfach zusätzlich ein Quartal in der Allgemeinmedizin oder einem anderen ambulanten Fachgebiet absolviert werden soll [1]. Diese grundlegende Veränderung in der medizinischen Ausbildung wird insbesondere Auswirkungen auf die chirurgischen Fachgebiete haben, sodass eine spezielle Vorbereitung essenziell ist.

Aktuell ist in der gesamten sog. westlichen Welt eine Abnahme der Attraktivität des chirurgischen Werdegangs für Medizinstudierende zu beobachten [3–5]. Die Ursachen hierfür sind sicherlich multifaktoriell. So haben sich die Ansprüche der Medizinstudierenden und angehenden Berufseinsteiger:innen an ihre zukünftige Arbeitsstätte gewandelt. Als Hauptgründe für die geringe Motivation, eine chirurgische Profession zu wählen, werden fehlende Planungssicherheit, eine unausgewogene Work-Life-Balance, eine ungenügende Kinderbetreuung sowie fehlende Flexibilität bei Arbeitszeiten und ein mangelnder respektvoller Umgangston genannt [6]. Insbesondere für die Ärzt:innen der Generation Y (Geburtsjahre 1981–2000) spielt eine ausgewogene Work-Life-Balance eine entscheidende Rolle bei der Wahl einer Arbeitsstelle [7].

Es bleibt interessant zu beobachten, wie sich der Eintritt der aktuellen Berufsanfänger:innen, die zur Generation Z zählen (geboren zwischen 1993 und 2012), auf den Arbeitsmarkt auswirken wird. Obwohl eine pauschale Verallgemeinerung stets schwierig ist, wird ihnen prognostiziert, eine starke Arbeitsmoral ähnlich der Babyboomer-Generation (Geburtsjahre 1947–1964) zu haben und gleichzeitig die Widerstandsfähigkeit der Generation X (geboren zwischen 1965–1985) zu besitzen [8].

Zum aktuellen Zeitpunkt ist der Beruf des Chirurgen/der Chirurgin unter anderem durch lange Arbeitszeiten, ein hohes persönliches Engagement und die Zurückstellung der eigenen Bedürfnisse zugunsten der Profession geprägt. Die praktische Ausbildung erfolgt größtenteils im Rahmen eines traditionellen „Meister-Schüler-Verhältnisses“, was potenziell durch subjektive zwischenmenschliche Beziehungen und strenge Hierarchien beeinflusst werden kann [6]. Diese Aspekte stehen jedoch im Widerspruch zur Selbstwahrnehmung und den Werten der heutigen Studierendengeneration, insbesondere der Generation Y und Z. Für diese Generation hängt die Auswahl einer Weiterbildungsstätte maßgeblich davon ab, welches Ausbildungsprogramm die individuellen beruflichen Ziele am besten unterstützt [8].

Darüber hinaus beginnen nur wenige Frauen ihre berufliche Laufbahn in der Chirurgie, obwohl die Mehrheit der aktuell Studierenden weiblich ist [9, 10]. Zudem ist die Zahl an Frauen, die ihre chirurgische Facharztweiterbildung vorzeitig abbrechen, hoch [11].

## Auswirkungen des Nachwuchsmangels

Aufgrund dieser Entwicklungen sehen sich die chirurgischen Fächer mit einem erheblichen Nachwuchsproblem konfrontiert, das sich bereits an vielen Orten deutlich zeigt. In einer landesweiten Umfrage gaben 80 % der befragten chirurgischen Chefärzte und (leitenden) Oberärzte an, dass sie einen spürbaren Mangel an Bewerber:innen wahrnehmen [7]. Von den Befragten bestätigten zusätzlich 94 %, qualitative Defizite bei den Bewerber:innenprofilen festzustellen. Hierdurch sahen sich 90 % der befragten Ärzte gezwungen, ihre eigenen Anforderungen soweit zu senken, dass ein unerwünschter Qualitätsverlust hingenommen werden musste. 88 % der Befragten äußerten die Sorge, dass sich der Bewerbermangel negativ auf die Versorgungsqualität in den deutschen chirurgischen Kliniken auswirken könnte [12].

Eine Folge dieses Prozesses ist die Entstehung eines ungewollt aggressiven Wettbewerbs zur Nachwuchsgewinnung, der sich nicht nur auf die Chirurgie beschränkt, sondern auf alle Fächer [13].

## Maßnahmen zur Nachwuchsgewinnung

Neben den sog. „Summer Schools“, die mittlerweile weit verbreitet sind und Fachgesellschaften sowie Kliniken zur Talentförderung, Rekrutierung und Imagepflege dienen, setzen Kliniken und einzelne Abteilungen verstärkt auf finanzielle Unterstützung in Form von Stipendien, um das Interesse der Medizinstudierenden zu gewinnen. Diese beinhalten im Gegenzug eine vertraglich zugesicherte Verpflichtung, nach Abschluss des Studiums eine festgeschriebene Zeit in der jeweiligen Klinik oder Abteilung zu arbeiten [13, 14].

Diese Idee wurde weiter ausgebaut und in Nordrhein-Westfalen 2018 das sog. „Landarztgesetz“ eingeführt, um dem Mangel an Hausärzt:innen in ländlichen Regionen entgegenzuwirken. Im Rahmen dieses Gesetzes werden jedes Semester eine feste Anzahl von Studienplätzen an Bewerberinnen und Bewerber vergeben, die sich im Gegenzug dazu verpflichten, nach Abschluss ihres Studiums eine Weiterbildung zu absolvieren, die sie zur Tätigkeit als Hausärztin oder Hausarzt berechtigt. Des Weiteren müssen sie sich für einen Zeitraum von 10 Jahren in Gebieten niederlassen, in denen ein besonderer Bedarf an hausärztlicher Versorgung besteht [13]. Derartige Ansätze gibt es für die Chirurgie bisher noch nicht, sollten aber von den Fachverbänden insbesondere für Kliniken außerhalb der Ballungszentren bzw. in strukturschwachen Regionen in Betracht gezogen werden.

Zusätzlich zu den bereits erwähnten Ansätzen zur Nachwuchsgewinnung bedarf es weiterer Optionen, die speziell auf die Tatsache eingehen müssen, dass die überwiegende Mehrheit der Absolvent:innen des Medizinstudiums weiblich ist. Bislang wurde diesem Sachverhalt in den chirurgischen Fächern nur begrenzt Aufmerksamkeit geschenkt [9, 10].

So werden gezielte Programme zur Frauenförderung benötigt, um insbesondere weibliche Studierende anzuwerben, aber auch um bereits in der Weiterbildung befindliche Mitarbeiterinnen zu halten und ihnen eine gute Perspektive in der Chirurgie zu geben.

Hier müssen Konzepte entwickelt werden, die die Vereinbarkeit von Familie und Karriere sowohl für Ärztinnen als auch Ärzte ermöglichen, um den Verlust hochqualifizierten Personals langfristig zu vermeiden. Dies zwingt Kliniken umsetzbare Lösungen zu entwickeln, die eine operative Tätigkeit während der Schwangerschaft ermöglichen und flexible Teilzeitarbeitsmodelle unterstützen, ohne dass dadurch die Karriereentwicklung beeinträchtigt wird [11]. Hierbei zählen insbesondere operative Ausbildungskonzepte, die in einem Teilzeitmodell für Assistenzärztinnen und -ärzte realistisch durchführbar sind. Die Unterstützung bei der Suche nach geeigneter Kinderbetreuung spielt ebenfalls eine zentrale Rolle, um Mitarbeiter:innen bei Bedarf einen reibungslosen Wiedereinstieg zu ermöglichen.

## Optionen in der Lehre

Neben diesen Maßnahmen muss ein besonderes Augenmerk auf Lösungsansätze gelegt werden, die das Studium chirurgischen Abteilungen bietet. Zwar wird in vielen Krankenhäusern die chirurgische Lehre durch eine steigende Arbeitsbelastung des medizinischen Personals beeinträchtigt, jedoch werden gleichzeitig Modelle entwickelt, bei denen beispielsweise Stellen für studentische Hilfskräfte im Operationssaal genutzt werden, um Studierende für die Chirurgie zu begeistern. Diese Gelegenheiten stehen neben dem Praktischen Jahr und freiwilligen Famulaturen zur Verfügung, ohne dass langfristige vertragliche Verpflichtungen eingegangen werden müssen [13, 15].

Die Bemühungen chirurgischer Fachgesellschaften und Kliniken bei der Gewinnung medizinischen Personals konzentrieren sich häufig ausschließlich auf die späten Phasen des Studiums, insbesondere auf das Praktische Jahr [13]. Es gibt keine Pflichtfamulatur in der Chirurgie, sodass einige Studierende erst im Pflichttertial des Praktischen Jahres für einen längeren Zeitraum mit der Chirurgie in Berührung kommen.

Aus unserer Sicht ist es unabdingbar, dieser Situation bereits früh im Medizinstudium durch innovative Lehrkonzepte entgegenzuwirken. Es ist bekannt, dass die während der klinischen Ausbildung gesammelten Erfahrungen von Studierenden einen essenziellen Einfluss auf ihre spätere Berufswahl haben. Diese können zum Vorteil der chirurgischen Fächer beeinflusst werden [16, 17]. Laut den Ergebnissen des bayrischen Absolventenpanels entschieden sich innerhalb eines Jahres nach Abschluss des Medizinstudiums nur eine Minderheit von Absolventinnen und Absolventen, nämlich weniger als 10 %, für eine Spezialisierung in einem chirurgischen Fachgebiet [18]. Gleich mehrere Studien können ein abnehmendes Interesse für eine chirurgische Weiterbildung im Verlauf des Studiums aufzeigen, obwohl zu Beginn des Studiums für über 40–60 % der Studierenden ein chirurgischer Werdegang vorstellbar ist [13, 15].

Hierbei ist zu bedenken, dass durch die neue Approbationsordnung der Schwerpunkt auf die allgemeinmedizinische und ambulante Versorgung gelegt werden soll und somit für andere Fächer, wie die Chirurgie, mit klassischen Lehrkonzepten wie Vorlesungen noch weniger Zeit bleiben wird, um sich positiv zu präsentieren und „in die Tiefe des Faches zu gehen“.

Bereits jetzt werden innovative Lehr- und Prüfungsformate von den Studierenden der aktuellen Generation aktiv gefordert und von den Lehrenden verlangt, die Ausarbeitung der Lehrveranstaltungen nicht mehr als rein frontalen Unterricht zu verstehen, sondern eine aktive Einbindung der Studierenden angemessen zu fördern und fordern [19, 20].

Studierende müssen sich immer mehr theoretisches Wissen aneignen [21]. Verdoppelten sich wissenschaftliche Erkenntnisse seit Ende des Zweiten Weltkrieges alle 25 Jahre, geschieht dies heute im klinischen Bereich alle 18 Monate. Laut IBM könnte sich dieses Wissen mit der Ausweitung des Internets sogar alle 12 h verdoppeln [22]. Allein im Jahr 1997 wurden jährlich mehr als 6 Mio. medizinische Artikel veröffentlicht. Mit den heutzutage frei zugänglichen Zeitschriften, selbst veröffentlichten Büchern sowie unzähligen Internetquellen ist es sowohl für die Lehrenden als auch die Lernenden quasi unmöglich, stets auf dem neuesten Stand zu bleiben [8]. Hier gilt es für die Lehrenden in der Chirurgie, eine ausgewogene Balance zwischen Theorie und Praxis zu erreichen. Aktuell bestehen Defizite in der Ausbildung insbesondere im Hinblick auf praktische Fähigkeiten, sodass sich Berufsanfänger:innen durch das Studium nicht ausreichend auf die praktischen Anforderungen im Beruf vorbereitet fühlen [23, 24].

## Unsere Maßnahme gegen den Nachwuchsmangel in der Chirurgie – der „Surgical Track“

Es ist daher notwendig, die Lehre chirurgisch-praktischer Fertigkeiten frühzeitig in das Kurrikulum zu integrieren, so wie es der Masterplan 2020 vorsieht, um sowohl vertikale als auch longitudinale Lehransätze zu fördern und dadurch das Interesse der Studierenden für die Chirurgie bereits zu einem frühen Zeitpunkt des Studiums zu wecken [5, 25]. Aus diesem Grund haben wir den „Surgical Track“ entwickelt, der gezielt innovative Lehrkonzepte anbieten soll, um Studierende frühzeitig für das Fach Chirurgie zu begeistern und innovative Lehrkonzepte bis in die Assistenzarztzeit zu tragen.

Dieser fundiert auf zwei Säulen, auf die wir im Folgenden näher eingehen wollen.

**Figure 1:**
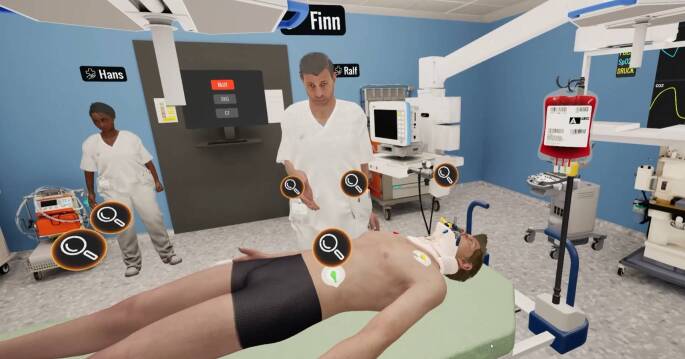

Die erste dieser Säulen basiert auf Virtual-Reality(VR)-gestützten Simulationen. Hier können Studierende z. B. in der Vorklinik anhand anatomischer Modelle virtuelle Operationen durchführen, um sich die topographische Anatomie besser veranschaulichen zu können. Zudem wurden Simulationen kreiert, die Notfallszenarien im Schockraum oder auf der Normalstation trainieren lassen können. So können die Abläufe bei kritisch kranken Patienten mit z. B. einer Lungenembolie bzw. einem Myokardinfarkt oder auch einer Milzruptur mit mehreren Spieler:innen in einem Multiplayer-Szenario erlernt werden (Abb. 1).

Die Gründe, VR-basierte Simulationen zu benutzen, sind vielfältig. Simulatoren werden mehr und mehr sowohl in der professionellen Praxis als auch in der medizinischen Ausbildung benutzt. Eine leicht zugängliche Computertechnologie ermöglicht, VR-basierte Kurse in das medizinische Kurrikulum einzubauen [26]. Simulationen bieten die Möglichkeit, ausgewählte Fähigkeiten, Notfallsituationen, seltene Ereignisse oder Extremsituationen in einem geschützten Umfeld zu erlernen. Hier gemachte Fehler haben keine Konsequenzen für die Studierenden oder reale Patient:innen, können analysiert und durch Wiederholungen vermieden werden [26–28]. Hierbei ist der große Vorteil der VR-Simulation, eine immersive Spielerfahrung zu erzeugen, wodurch realitätsnahe Trainingsszenarien möglich sind [29]. Die kommerzielle Entwicklung solch hochwertiger Produkte ist jedoch ressourcenintensiv und subventionsbedürftig, da durch die Entwicklungskosten hohe Verkaufszahlen notwendig wären, um profitabel zu sein [30]. Des Weiteren ist der Erfolg solcher Programme nicht nur von der technischen Qualität allein, sondern auch davon abhängig, wie viel Lerninhalt vermittelt wird. Kommerzielle Programme enthalten häufig fehlerhafte medizinische Inhalte bzw. haben einen Fokus, der aus kurrikularer Sicht limitiert ist [31]. Aus diesem Grund ist es wünschenswert, dass die Entwicklung solcher Lehrmethoden in universitären Händen bleibt, da diese ein Hauptantreiber für innovative Lehrmethoden sein sollen [32].

Die zweite Säule des Surgical Tracks konzentriert sich darauf, neue Technologien wie die Robotik in die studentische Ausbildung einzubringen. Dadurch sollen die Studierenden frühzeitig mit den für ihre zukünftige Karriere als Chirurg:innen relevanten Technologien vertraut gemacht werden. Wir erweitern und ergänzen die bestehenden Ausbildungsmodule sinnvoll, indem wir sowohl Simulationen als auch praktische Übungen mit Robotern in den Lehrplan integrieren. Durch diese Maßnahmen ermöglichen wir den Studierenden, sich umfassende Fähigkeiten im Umgang mit den neuesten Technologien anzueignen, die in der chirurgischen Praxis immer wichtiger werden. Da auch erfahrene Chirurg:innen die Robotertechnik meist zunächst komplett in VR erlernen, bevor diese im Operationssaal angewendet wird, lässt sich dieser Ansatz problemlos auf Studierende übertragen.

In den letzten Jahren hat die Anwendung robotergestützter Eingriffe in der Viszeralchirurgie weltweit signifikant zugenommen, ähnlich wie es zu Beginn der 1990er-Jahre bei der Einführung der Laparoskopie der Fall war. Dies ist auf verschiedene Faktoren zurückzuführen, darunter die gestiegene Nachfrage seitens der Patientinnen und Patienten sowie eine Preisreduktionen aufgrund des zunehmenden Wettbewerbs unter den Anbietern. Dennoch sind vor allem medizinische Gründe ausschlaggebend für diese Entwicklung.

Zahlreiche Studien haben bereits gezeigt, dass die Robotik durch eine verbesserte, hoch auflösende, dreidimensionale Sicht auf den Operationsbereich, einen Ausgleich leichter Zitterbewegungen durch die Robotikkonsole und eine Erhöhung der Bewegungsfreiräume bzw. eine verbesserte Angulation der Laparoskopie ebenbürtig bzw. überlegen ist [33–35].

Durch die Integration der Robotik in das medizinische Kurrikulum besteht die Möglichkeit, die Faszination dieser Technologie zu nutzen, um das Interesse an den chirurgischen Fächern zu steigern.

**Figure 2:**
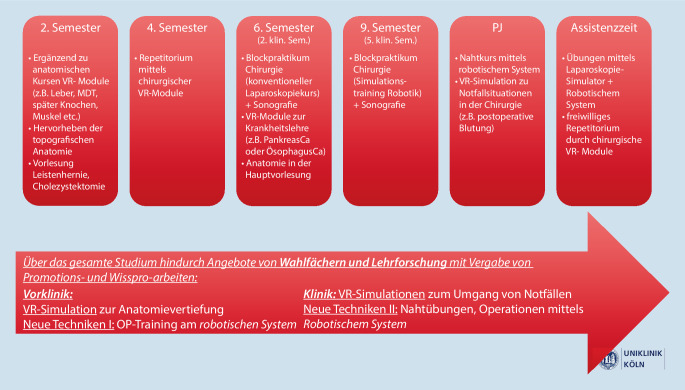

Der gesamte Surgical Track wird in Abb. 2 zusammengefasst dargestellt.

**Figure 3:**
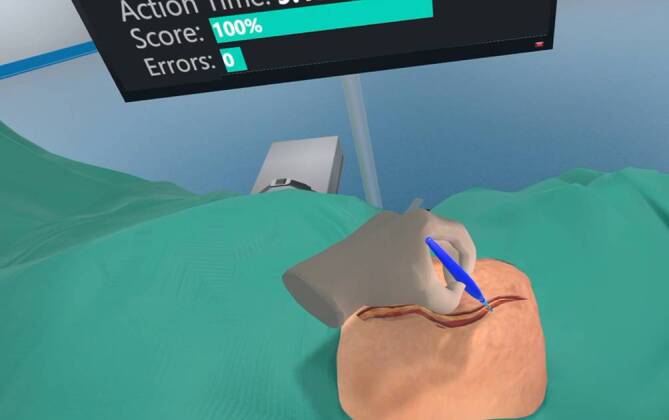

Wie in Abb. 2 gezeigt, beginnt der Surgical Track bereits im 2. Semester des Studiums. In diesem Abschnitt werden anatomische VR-Modelle gezielt eingesetzt, um die anatomischen Kurse sinnvoll zu ergänzen. Gleichzeitig werden einfache topografische Operationstrainingsmodule (Abb. 3) verwendet, um den Studierenden die Relevanz der Anatomie für künftige klinische und operative Tätigkeitsfelder bewusst zu machen. Zudem wird bereits im 2. Semester im Sinne des Masterplans 2020 eine Vorlesung über Leistenhernien gehalten und mittels VR-Simulationen ergänzt.

Im 4. Semester können die chirurgischen VR-Module als Repetitorium genutzt werden. Im 6. Semester werden die Module (konventionelle Laparoskopie und Sonographie) mit VR-Modulen zum Pankreas- und Ösophaguskarzinom ergänzt und so die Zeiten von Frontalunterricht weiter reduziert. Auch findet in diesem Semester unsere Hauptvorlesung statt. Im 9. Semester wird unser Blockpraktikum durch ein robotisches Simulationstraining ergänzt. Im Praktischen Jahr bieten wir für unsere Studierenden gezielt Nahtkurse mittels robotischen Systemen sowie Kurse an, in denen Notfallsituationen in VR simuliert und trainiert werden. In der Assistenzzeit werden diese Kurse dann weiter ausgebaut, sodass wir hier in regelmäßigen Abständen Laparoskopie‑, Robotik- und VR-Notfall-Trainingseinheiten anbieten können. Sowohl die VR-basierten Simulationen als auch die Robotiktrainingseinheiten sind jeweils in einem Lehransatz integriert, der an den Wissensstand der Studierenden oder Assistenzärzt:innen angepasst ist und theoretische, praktische und reflektierende Abschnitte enthält.

Durch zusätzliche Angebote von Wahlfächern und begleitenden wissenschaftlichen Lehrprojekten (wissenschaftliche Projekte und Promotionsarbeiten) können für Studierende im Rahmen des Surgical Tracks weitere Anreize geschaffen werden, sich tiefer mit chirurgischen Themen zu befassen.

## Aktueller Stand und Erkenntnisse aus den ersten Anwendungen

**Figure 4:**
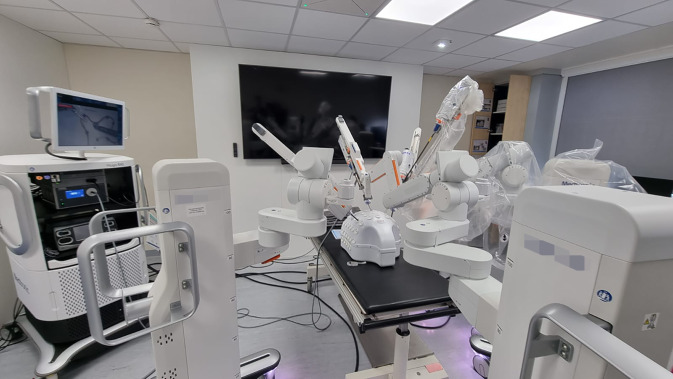

In Teilen kam der in Abb. 2 dargestellte Surgical Track im Sommersemester 2023 erstmals zur Anwendung. Dabei wurde primär das Hauptaugenmerk auf die Optimierung der VR-Programme und der technischen Schnittstellen gelegt. 108 Studierende konnten bereits im Zuge unseres chirurgischen Blockpraktikums Erfahrungen in der robotischen Chirurgie mittels des Hugo-RAS-Systems von Medtronic (Dublin, Irland) sammeln (Abb. 4). Neben den erhaltenen Optimierungsvorschlägen konnte bei allen Teilnehmenden eine hohe Zufriedenheit bei den Lehreinheiten festgestellt werden. Zudem haben die Studierenden den Wunsch geäußert, dass mehr derartiger Module im Studium integriert werden sollen. Eine ausführliche Evaluation des Kurrikulums ist in den kommenden Jahren zu erwarten. Die hier gewonnen Erkenntnisse können für Dozierende von Interesse sein, um sich mit den Herausforderungen, Gestaltung und Implementierung solch eines Kurrikulums vertraut zu machen.

## Fazit

Auch wenn die Gründe für den Nachwuchsmangel in der Chirurgie vielschichtig sind, kann durch qualitativ hochwertige und innovative Lehre positiv für unser Fach geworben werden. Ausbildungskonzepte wie der Surgical Track bieten zusätzlich eine Möglichkeit, die Wertschätzung für eine engagierte Lehre zu fördern, auch wenn der tatsächliche Nutzen derartiger weitreichenden Modelle erst durch Langzeitstudien und Evaluationen eruiert werden kann. Innovative und neue Zukunftstechnologien wie die VR und die Robotik bergen zudem das Potenzial, die Neugier an der Chirurgie bereits früh im Studium zu wecken.

Durch die einzigartige Kombination aus virtuellen Simulationen, robotischer Chirurgie und innovativen Lehrkonzepten erhalten die Studierenden an unserem Standort eine zukunftsfähige und umfassende Vermittlung von Lehrinhalten im Bereich der Viszeralchirurgie. Dadurch haben sie die Möglichkeit, innovative Technologien nicht nur theoretisch zu begreifen, sondern von Anfang an praktisch zu erleben. Wir erhoffen uns, dass diese Erfahrung in Zukunft nicht nur der Viszeralchirurgie, sondern auch anderen operativ tätigen Fächern wie der Orthopädie/Unfallchirurgie, Urologie oder Gynäkologie zugutekommen wird.

## Ausblick

**Figure 5:**
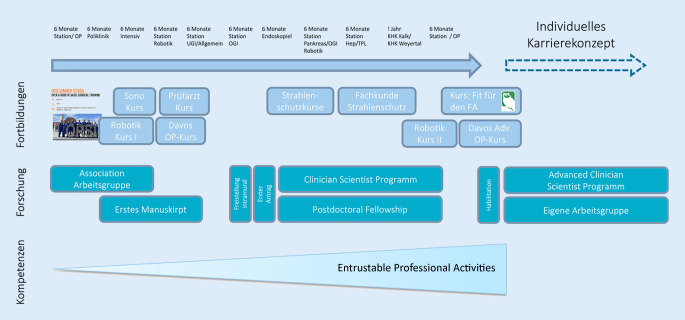

Die individuelle Karriereförderung sollte auch nach dem Studium nicht aufhören. Durch klare Strukturen in der Weiterbildung der Assistenzärzt:innen müssen sowohl operative als auch individuelle Karriereziele bis zum Facharzt/Fachärztin und darüber hinaus bestmöglich gefördert werden (Abb. 5).

Beispiele wie die von der Jungen Chirurgie veröffentlichten Teilschritte oder modulare Weiterbildungskonzepte mit auch klaren Strukturen für die wissenschaftliche Entwicklung sollten hierbei Berücksichtigung finden [36].
